# Assessing the Pre-Analytical Stability of Small-Molecule Metabolites in Cerebrospinal Fluid Using Direct-Infusion Metabolomics

**DOI:** 10.3390/metabo9100236

**Published:** 2019-10-18

**Authors:** Hanneke A. Haijes, Eline A.J. Willemse, Johan Gerrits, Wiesje M. van der Flier, Charlotte E. Teunissen, Nanda M. Verhoeven-Duif, Judith J.M. Jans

**Affiliations:** 1 Section Metabolic Diagnostics, Department of Genetics, University Medical Center Utrecht, Utrecht University, Lundlaan 6, 3584 EA Utrecht, The Netherlands; j.gerrits@umcutrecht.nl (J.G.); N.Verhoeven@umcutrecht.nl (N.M.V.-D.); 2 Section Metabolic Diseases, Department of Child Health, University Medical Center Utrecht, Utrecht University, Lundlaan 6, 3584 EA Utrecht, The Netherlands; 3 Neurochemistry Laboratory and Biobank, Department of Clinical Chemistry, Amsterdam Neuroscience, Amsterdam University Medical Center, Vrije Universiteit Amsterdam, De Boelelaan 1117, 1081 HV Amsterdam, The Netherlandsc.teunissen@amsterdamumc.nl (C.E.T.); 4 Alzheimer Center and Department of Neurology, Amsterdam Neuroscience, Amsterdam University Medical Center, Vrije Universiteit Amsterdam, De Boelelaan 1117, 1081 HV Amsterdam, The Netherlands; wm.vdflier@amsterdamumc.nl; 5 Department of Epidemiology and Biostatistics, Amsterdam University Medical Center, Vrije Universiteit Amsterdam, De Boelelaan 1117, 1081 HV Amsterdam, The Netherlands

**Keywords:** biomarker stability, pre-analytical storage conditions, small-molecule metabolites, cerebrospinal fluid, metabolomics, direct-infusion mass spectrometry, DIMS, neurometabolic diagnostics

## Abstract

Metabolomics studies aiming to find biomarkers frequently make use of historical or multicenter cohorts. These samples often have different pre-analytical conditions that potentially affect metabolite concentrations. We studied the effect of different storage conditions on the stability of small-molecule metabolites in cerebrospinal fluid to aid a reliable interpretation of metabolomics data. Three cerebrospinal fluid pools were prepared from surplus samples from the Amsterdam Dementia Cohort biobank. Aliquoted pools were exposed to different storage conditions to assess the temperature and freeze/thaw stability before final storage at −80 °C: storage up to four months at −20 °C and up to one week at either 5–8 °C or 18–22 °C and exposure to up to seven freeze/thaw cycles. Direct-infusion high-resolution mass spectrometry was performed, resulting in the identification of 1852 *m*/*z* peaks. To test the storage stability, principal component analyses, repeated measures analysis of variance, Kruskal–Wallis tests, and fold change analyses were performed, all demonstrating that small-molecule metabolites in the cerebrospinal fluid (CSF) are relatively unaffected by 1–3 freeze/thaw cycles, by storage at −20 °C up to two months, by storage at 5–8 °C for up to 72 h, or by storage at 18–22 °C for up to 8 h. This suggests that these differences do not affect the interpretation of potential small-molecule biomarkers in multicenter or historical cohorts and implies that these cohorts are suitable for biomarker studies.

## 1. Introduction

The cerebrospinal fluid (CSF) is the closest possible read-out of all body fluids of metabolite concentrations in the brain, as it circulates in the subarachnoidal space and is in direct contact with the brain parenchyma, meninges, and spinal cord [1]. For this reason, CSF has been studied to identify biomarkers to aid diagnostics for a variety of neurological disorders, from neurodegenerative diseases and cerebral infections to inborn errors of metabolism.

Since studies aiming to identify biomarkers generally require large sample sizes, they frequently make use of historical or multicenter cohorts. However, the pre-analytical conditions in these cohorts, including the collection, processing, and storage of these samples, often vary [1]. These differences potentially affect the metabolite concentrations, resulting in bias [1]. Whereas studies on the effects of pre-analytical conditions on CSF often focus on protein stability [1], only a few studies have focused on the stability of small-molecule metabolites [2,3,4]. For example, one study found that the CSF concentrations of four amino acids (glutamic acid, glycine, aspartic acid, and taurine) were not affected by storage at −20 °C or −80 °C, provided that the sample was deproteinized and neutralized before storage [2]. Another study demonstrated that storage for 72 h at room temperature, compared to immediate storage at −70 °C, resulted in decreased levels of citrate and increased levels of lactate, glutamine, creatine, and creatinine, whereas the levels of myo-inositol, glucose, pyruvate, acetate, and alanine were unaffected [3]. Others showed that storage for 30 or 120 min at room temperature, compared to immediate storage at −80 °C, significantly increased the concentrations of 10/17 amino acids measured in porcine CSF [4]. In addition, the majority of other measured small-molecule metabolites (*n* = 79) in this study [4] also demonstrated increased concentrations after 30 min and/or 120 min at room temperature, indicating that residual enzymatic activity in CSF after collection may lead to altered metabolite levels, possibly by the degradation of proteins [4], indicating that the storage conditions indeed affect the stability of small-molecule metabolites in CSF.

Although the studies performed to date give some indication of the potential effects of differences in pre-analytical storage conditions on the stability of small-molecule metabolites, only a limited number of metabolites were included in these studies, while targeted and untargeted metabolomics studies can potentially identify many more small-molecule metabolites. Thus, as targeted and untargeted metabolomics studies are more frequently performed for the identification of biomarkers in CSF, we studied the effect of different storage conditions on the stability of small-molecule metabolites in CSF to aid a reliable interpretation of metabolomics data.

## 2. Results

**Figure 1:**
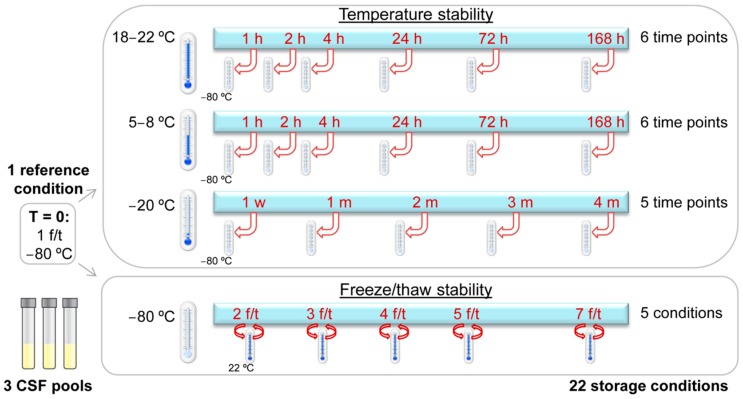
Overview of the different storage conditions analyzed to assess the temperature and freeze/thaw stability of small-molecule metabolites in cerebrospinal fluid. Three pools of cerebrospinal fluid were analyzed. For each pool, one reference sample and 22 samples for the different storage conditions were analyzed. Abbreviations: CSF: cerebrospinal fluid, f/t: freeze/thaw cycle, h: hours, m: months, w: week.

Untargeted metabolomics using direct-infusion high-resolution mass spectrometry resulted in a list of 1852 unique mass over charge (*m*/*z*) peaks, corresponding to 3806 unique annotations of metabolites that can occur endogenously, including isomers. This was in line with previous analyses [5,6]. In four out of 69 samples, direct infusion was hampered, resulting in the exclusion of the following samples: one of freeze/thaw cycle 1 (second CSF pool), one of freeze/thaw cycle 7 (first CSF pool), one of 5–8 °C for 168 h, and one of 18–22 °C for 2 h (both in the third CSF pool). In total, 65/69 samples were used for statistical analyses. An overview of the different pre-analytical storage conditions assessed is given in Figure 1.

### 2.1. Analysis of Variability.

To assess which part of the identified variability is solely due to technical aspects of the analysis, the variability within the run was monitored by the addition of stable isotope-labeled compounds (sILC) to each sample during the sample preparation. The median coefficient of variation of the sILCs was 0.166 (95% confidence interval: 0.127–1.565), in line with previous analyses [5] (Table 1).

As the sILCs were added during the sample preparation, the median absolute variation of the most extreme storage conditions was calculated as a reflection of the variation in the sample that can be solely attributed to analysis variability, and not to variability due to the different storage conditions tested. The median absolute variation of the sILCs was 0.384, 0.072, 0.171, and 0.188 for the samples that experienced the most extreme storage conditions: freeze/thaw cycle 7, storage at –20 °C for four months, storage at 5–8 °C for 168 h, and storage at 18–22 °C for 168 h, respectively (Table 1). 

### 2.2. Freeze/Thaw Stability

To assess whether the metabolome changed due to multiple freeze/thaw cycles, principal component analysis was performed for the 1852 *m*/*z* peaks as well as for the selection of 106 *m*/*z* peaks corresponding to metabolites marked as important for neurometabolic diagnostics (Table 2). This did not reveal any clustering of the different freeze/thaw cycles (Figure S1), indicating that the metabolome as a whole did not change upon multiple freeze/thaw cycles. In addition, no clustering of the samples from the three different CSF pools was observed, suggesting that the three CSF pools had comparable metabolomes (Figure S1).

Repeated measures analysis of variance (ANOVA) assessing all 1852 *m*/*z* peaks for all freeze/thaw cycles revealed no significant differences; nor did Kruskal–Wallis tests comparing freeze/thaw cycle 7 to freeze/thaw cycle 1. When assessing the selection of 106 *m*/*z* peaks corresponding to neurometabolic metabolites, neither the repeated measures ANOVA comparing all freeze/thaw cycles nor the Kruskal–Wallis tests comparing freeze/thaw cycle 7 to freeze/thaw cycle 1 revealed any significant differences, suggesting that the stability of individual *m*/*z* peaks is unaffected by multiple freeze/thaw cycles.

**Figure 2:**
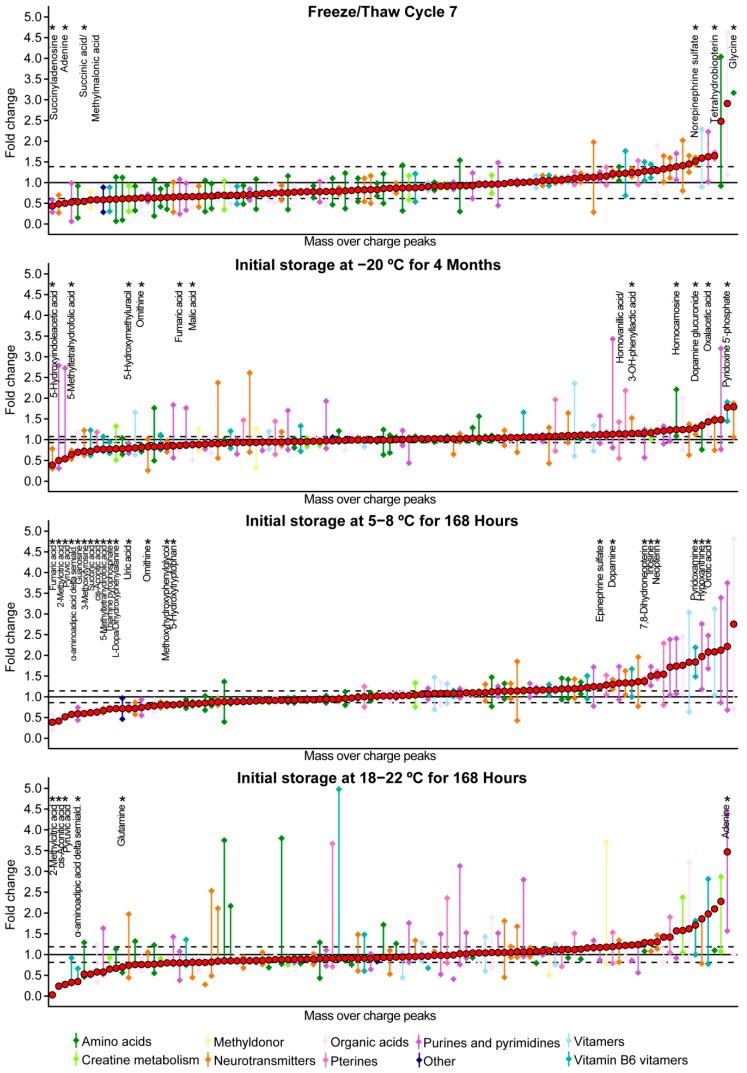
*m*/*z* peaks corresponding to metabolites important for neurometabolic diagnostics that are possibly affected by the most extreme storage conditions compared to the reference sample, after correction for the analysis variability. The y-axis depicts the fold change of the most extreme storage condition compared to the reference sample; the x-axis depicts the 106 *m*/*z* peaks corresponding to metabolites important for neurometabolic diagnostics (as listed in Table 2). Colored diamonds depict the upper and lower limit of the 95% confidence intervals; colored lines depict the range of the 95% confidence interval. Red filled circles depict the mean fold change. Colors depict the group of neurometabolic metabolites, as listed in the legend. The horizontal black line depicts fold change = 1, horizontal dashed lines indicate the range of the analysis variability: fold change plus or minus the median absolute variation of the stable isotope-labeled compounds for the analysis series. Asterisks depict the metabolites for which the lower limit of 95% confidence interval was above the upper dashed line and metabolites for which the upper limit of the 95% confidence interval was below the lower dashed line. For these, metabolite names are indicated below the asterisks. These metabolites correspond to the metabolites illustrated in Figures S2–S5.

To detect more subtle changes in metabolite stability, the freeze/thaw stability was further assessed by an analysis of fold changes (FC). After correction for the median absolute variation reflecting the analysis variability, 119 of the 1852 *m*/*z* peaks were considered to be affected by the most extreme storage condition, of which six were neurometabolic metabolites (Table 3, Figure 2). There was no specific group of neurometabolic metabolites that was more affected than another group (Figure 2). The six metabolites that were possibly affected by multiple freeze/thaw cycles were visually assessed (Figure S2). Remarkably, adenine concentrations showed a decrease of more than 50% after more than four freeze/thaw cycles and glycine concentrations showed a more than five-fold increase after more than five freeze/thaw cycles (Figure S2).

### 2.3. Temperature Stability

For all three temperature stability series, i.e., storage at −20 °C, at 5–8 °C or at 18–22 °C for different periods of time (Figure 1), principal component analysis, performed to assess whether the metabolome changed due to the storage conditions, did not reveal any clustering of the different time points (Figure S1), suggesting that the metabolome as a whole was not affected by the different storage conditions. In addition, no clustering of CSF pools was observed, indicating that the metabolic compositions of the pools was comparable (Figure S1).

Repeated measures ANOVA assessing all time points and Kruskal–Wallis tests comparing the most extreme time points to the reference samples revealed no significant differences. For the selection of 106 *m*/*z* peaks corresponding to neurometabolic metabolites, Kruskal–Wallis tests comparing the most extreme time points to the reference samples did not display any significant differences, and repeated measures ANOVA only disclosed a significant change in mass peak intensity for cis-aconitic acid when stored at 18–22 °C, suggesting that the stability of individual *m*/*z* peaks is unaffected by the different storage conditions, except for cis-aconitic acid when stored at 18–22 °C.

To detect more subtle changes, the temperature stability was further assessed by analysis of FCs. After correction for the median absolute variation reflecting the analysis variability, 276 of the 1852 *m*/*z* peaks were considered to be affected by storage for four months at −20 °C, 419 *m*/*z* peaks were considered to be affected by storage for a week at 5–8 °C and 121 *m*/*z* peaks were considered to be affected by storage for a week at 18–22 °C (Table 3). Of these peaks, 11, 23, and six (25 unique *m*/*z* peaks) were among the 106 *m*/*z* peaks corresponding to neurometabolic metabolites (Table 3, Figure 2). No specific group of neurometabolic metabolites was more affected than another group (Figure 2). Metabolites that were possibly affected by prolonged storage at −20 °C, 5–8 °C, and 18–22 °C were visually assessed (Figure S3–S5). 5-methyltetrahydrofolic acid concentrations were decreased after storage for more than two months at −20 °C and dopamine glucuronide concentrations were increased after storage for more than three months at −20 °C (Figure S3). Prolonged storage at 5–8 °C resulted in a decrease in cis-aconitic acid, pyruvic acid, 2-methylcitric acid, and α-aminoadipic acid delta-semialdehyde concentrations and an increase in orotic acid and neopterin concentrations (Figure S4). Lastly, prolonged storage at 18–22 °C resulted in a decrease in cis-aconitic acid, pyruvic acid, 2-methylcitric acid, α-aminoadipic acid delta-semialdehyde and glutamine concentrations, and in an increase in adenine concentrations (Figure S5).

## 3. Discussion

We studied the effect of different storage conditions on the stability of small-molecule metabolites in CSF to aid a reliable interpretation of metabolomics results. We present two main findings. First, the majority of small-molecule metabolites seems to be relatively unaffected by multiple freeze/thaw cycles, as clustering analysis as well as repeated measures ANOVA comparing the different conditions and Kruskal–Wallis tests comparing the most extreme condition to the reference sample revealed no significant differences in metabolite intensities. Analysis of FCs revealed that adenine levels decreased more than 50% after more than four freeze/thaw cycles, and that glycine levels showed a five-fold increase after more than five freeze/thaw cycles (Figure S2). Two studies reported stable glycine concentrations in CSF after three freeze/thaw cycles, but these studies did not investigate the effects of more freeze/thaw cycles on glycine concentrations [7,8]. Altogether, we conclude that different numbers of freeze/thaw cycles in a single cohort are not expected to influence levels of small-molecule metabolites, but we suggest reticence in interpretation of potential biomarker concentrations in samples with more than four freeze/thaw cycles, especially with respect to adenine and glycine.

The second main finding is that the majority of small-molecule metabolites seems relatively unaffected by the time and temperature at which they were stored. For temperature stability analyses, clustering analysis as well as repeated measures ANOVA and Kruskal–Wallis tests revealed no significant differences, except for cis-aconitic acid when stored at 18–22 °C. Analysis of FCs revealed that the levels of some metabolites, including 5-methyltetrahydrofolic acid, dopamine glucuronide, cis-aconitic acid, pyruvic acid, 2-methylcitric acid, α-aminoadipic acid delta-semialdehyde, orotic acid, neopterin, glutamine and adenine might be affected by prolonged storage at −20 °C, 5–8 °C, or 18–22 °C (Figure S3–S5). In another study, our group investigated the stability of 5-methyltetrahydrofolic acid in CSF samples and demonstrated decreased concentrations after a week of storage at room temperature, but, contrasting with our findings in this study, there were stable levels after storage for a week at 4 °C [9]. In the study previously performed by our group, storage at −20 °C was not included [9]. Another group found an increase of glutamine at 18–22 °C [3], whereas we report a decrease of glutamine levels at this time point. Moreover, we did not observe decreased levels of citrate, nor increased levels of lactate, creatine, and creatinine. We observed decreased levels of pyruvic acid when stored at 18–22 °C, while Levine et al. reported stable levels of pyruvic avid [3]. Although Levine et al. used proton NMR analyses to detect potential differences, there were no other important differences in the study design that could explain these differences. Thus, the effects of prolonged storage at −20 °C, 5–8 °C, or 18–22 °C before final storage at −80 °C are debatable. We suggest being cautious by including samples stored at −20 °C for more than two months (Figure S3), samples stored at 5–8 °C for more than 72 h (Figure S4), and samples stored at 18–22 °C for more than 8 h (Figure S5) in a cohort study for biomarker identification.

There are a few limitations to this study. First, while a wide range of pre-analytical conditions was studied, the sample size per condition was relatively small (*n* = 3), hampering the power of the analysis. For this reason, the relative stability of small-molecule metabolites in CSF might be overestimated, as biologically relevant changes in metabolite concentrations might not have reached statistical significance. Second, due to the exploratory nature of this study, surplus CSF samples were used that needed an extra thawing cycle for pooling. The reference sample thus underwent one freeze/thaw cycle instead of zero freeze/thaw cycles, hampering the interpretation of the effects of one freeze/thaw cycle on the concentrations of small-molecule metabolites.

Despite these limitations, an important strength of this study is that multiple statistical analyses were performed, including unsupervised clustering analysis, repeated measures ANOVA for comparing all storage conditions for each identified *m*/*z* peak, Kruskal–Wallis tests for comparing the most extreme storage condition to the reference sample for each identified *m*/*z* peak, and analysis of FCs with correction for the analysis variability to assess the more subtle differences in mass peak intensities. All these analyses pointed towards the same conclusion: concentrations of small-molecule metabolites are relatively unaffected by most pre-analytical storage conditions. Moreover, the nature of this study was untargeted, hypothesis-free, and broad, as the stability of more than 1800 *m*/*z* peaks was tested.

## 4. Materials and Methods

### 4.1. Sample Collection and Storage

Surplus CSF samples from the Amsterdam Dementia Cohort (ADC) biobank (e.g., samples having incomplete clinical information for use in clinical validation studies), collected between 2000 and 2017, were used [10]. For the ADC biobank, CSF was collected by lumbar puncture in 10-mL polypropylene tubes (Sarstedt, Nümbrecht, Germany) and centrifuged at 1800–2100× *g* for 10 min at 4 °C within 2 h, using a predefined procedure, according to international guidelines [11]. The CSF was divided into polypropylene tubes (1.5 or 2.0 mL; Sarstedt) in 500 µL volumes and was stored at −80 °C [10,11].

The biobanking protocol of the ADC was approved by the review board of the Vrije Universiteit Medical Center of Amsterdam. All procedures followed were in accordance with the ethical standards of the institution and with the Helsinki Declaration of 1975, as revised in 2000. All subjects provided written consent.

Aliquots of 0.5 mL per sample from multiple CSF samples from three to six different individuals were merged into a CSF pool in 50-mL polypropylene tubes. In total, three CSF pools with different compositions were acquired. Aliquots of 500 µL of these three CSF pools were stored in 1.5-mL polypropylene tubes with screw caps (Sarstedt). The aliquoted CSF pools were exposed to storage conditions as described in the standard operating procedure for sample stability [12] and as depicted in Figure 1, i.e., to assess the temperature stability of storage for up to four months at −20 °C and up to one week at either 5–8 °C or 18–22 °C before final storage at −80°C; and to assess freeze/thaw stability exposure to up to seven freeze/thaw cycles (Figure 1). One reference aliquot of each pool was stored at −80 °C directly at time point 0. This sample had thus undergone one freeze/thaw cycle.

### 4.2. Sample Preparation and Analysis

Metabolomics analysis of CSF was performed as previously described by our group [5,6]. Samples were thawed to room temperature. Five microliters of the sample were added to 140 μL of a working solution containing sILC, with fixed concentrations as previously described [6]. The solution was centrifuged for five minutes at 17,000× *g* and 105 μL of supernatant was diluted with 45 μL 0.3% formic acid (Emsure, Darmstadt, Germany). The solution was filtered using a methanol preconditioned 96-well filter plate (Acro prep, 0.2 μm GHP, NTRL, 1 mL well; Pall Corporation, Ann Arbor, MI, USA) and a vacuum manifold. The sample filtrate was collected in a 96-well plate (Advion, Ithaca, NY, USA). Direct-infusion high-resolution mass spectrometry was performed using a TriVersa NanoMate system (Advion), controlled by Chipsoft software (version 8.3.3, Advion), that was mounted onto the interface of a Q-Exactive high-resolution mass spectrometer (Thermo Scientific™, Bremen, Germany), with a scan range of 70–600 *m*/*z* [6]. For each sample, technical triplicates were analyzed, infusing each sample three times into the mass spectrometer. Samples were analyzed in a randomized order.

### 4.3. Data Processing

Data acquisition and processing were performed using a peak calling pipeline developed in the R programming language [5,6]. In this pipeline, mass peak identification and annotation was conducted by matching the *m*/*z* value of the mass peak with a range of two parts per million to metabolite masses present in the Human Metabolome Database, version 3.6 [13]. According to the Metabolomics Standards Initiative, the level of certainty of metabolite annotation is 2, as we putatively annotate compounds based on the matched *m*/*z* value of the mass peak [14]. Taking into account isomers and adduct ions, ~60,000 *m*/*z* peaks could be annotated with one or more possible annotations [5]. Metabolite annotations without adduct ions in negative or positive mode ([M − H]^−^, [M + H]^+^) or with single adduct ions [M + Na]^+^, [M + K]^+^ and [M + Cl]^−^ were selected. For each sample, intensities of the five selected *m*/*z* peaks were added together, resulting in one summed *m*/*z* peak intensity per metabolite annotation: ~6600 summed *m*/*z* peaks in total, per sample. Endogenous metabolite annotations and metabolite annotations with unknown function were selected [5]. No normalization of mass peak intensities was performed. The R code is available online (https://github.com/UMCUGenetics/DIMS).

### 4.4. Metabolite Groups

To study metabolites known or expected to be important in neurometabolic diagnostics in CSF in detail (www.metagene.de) [15], 106 *m*/*z* peaks corresponding to neurometabolic metabolites were selected and grouped into different categories (Table 2).

### 4.5. Data Analysis

To assess which part of the identified variability is due to technical aspects of the analysis, the variability within the run was monitored by the addition of sILC to each sample during the sample analysis. For each sILC, the coefficient of variation was calculated over all samples in the run by standard deviation intensity/mean intensity. To assess the most extreme conditions of each series, these samples were compared to the reference sample by calculating the median absolute variation of the sILC as follows: (1) calculation of the fold change: intensity of the most extreme condition/intensity of the reference sample; (2) fold change − 1; (3) conversion to absolute numbers; (4) calculation of the median absolute variation: median of the absolute variation of the three CSF pools.

For the total number of *m*/*z* peaks, as well as for the 106 *m*/*z* peaks corresponding to neurometabolic metabolites, for each series a principal component analysis was performed to visualize whether there was any clustering of different freeze/thaw cycles or time points, or of the three CSF pools. In addition, for each series a repeated measures ANOVA for all storage conditions was performed for each of the *m*/*z* peaks, as well as a non-parametric Kruskal–Wallis test comparing the most extreme condition to the reference sample for each of the *m*/*z* peaks. To control the familywise error rate in order to prevent any type I errors, all *p*-values were adjusted according to the Bonferroni method.

To assess which part of the identified variability is due to the different storage conditions, for each sample (except the reference sample), the FC was calculated as the intensity of the sample divided by the intensity of the reference sample. For each condition, the median absolute variation and 95% CI of the three CSF pools were calculated. Next, to correct for variability that can solely be attributed to the analysis, metabolites were defined as possibly affected by the storage condition when the lower limit of 95% CI was above the range attributed to the analysis variability (calculated by: FC 1.000 + median absolute variation of sILC), or when the upper limit of 95% CI was below the range attributed to the analysis variability (calculated by: FC 1.000 − median absolute variation of sILC). For each series, the median and 95% CI were plotted for the most extreme condition. This was performed for the total number of *m*/*z* peaks (data not shown), as well as for the 106 *m*/*z* peaks corresponding to neurometabolic metabolites. To assess whether mass peak intensities were affected in specific groups of neurometabolic metabolites, metabolites were colored according to the metabolite group (Table 2). Data analysis was performed in the R programming language.

## 5. Conclusions

In conclusion, we report that small-molecule metabolites in CSF are relatively unaffected by 1–3 freeze/thaw cycles, by storage at −20 °C for up to two months, by storage at 5–8 °C up to 72 h, or by storage at 18–22 °C up to 8 h, suggesting that minor differences in pre-analytical storage conditions in multicenter or historical cohorts used for the identification of biomarkers will not affect the interpretation of potential biomarkers, and implying that these cohorts are suitable for biomarker studies. We recommend caution when including samples with more extreme pre-analytical storage conditions. In addition, prior to using a biomarker for clinical decision-making, we recommend that the effects of pre-analytical conditions on the concentrations of any newly identified potential biomarker are tested.

## Figures and Tables

**Table 1:** Coefficient of variation of the analysis and median absolute variation of stable isotope-labeled compounds for the most extreme storage conditions.

Stable Isotope-Labeled Compound	CV	Median Absolute Variation			
		Freeze/Thaw Cycle 7	−20 °C, 4 Months	5–8 °C, 168 h	18–22 °C, 168 h
^15^N;2-^13^C-glycine	0.580	0.975	1.732	6.999	1.855
^2^H_4_-alanine	0.161	0.371	0.070	0.171	0.188
^2^H_3_-leucine	0.166	0.411	0.037	0.158	0.265
^2^H_3_-methionine	0.775	3.388	0.188	0.607	0.359
^13^C_6_-phenylalanine	0.149	0.374	0.050	0.142	0.204
^13^C_6_-tyrosine	0.150	0.349	0.043	0.098	0.206
^2^H_3_-aspartate	0.160	0.349	0.137	0.110	0.155
^2^H_3_-glutamate	0.126	0.247	0.099	0.110	0.074
^2^H_2_-ornithine	0.183	0.540	0.014	0.108	0.168
^2^H_2_-citrulline	0.134	0.231	0.070	0.131	0.038
^2^H_4_;^13^C-arginine	0.163	0.380	0.105	0.190	0.170
^2^H_8_-valine	0.155	0.377	0.042	0.124	0.222
^2^H_9_-carnitine	0.202	0.580	0.072	0.179	0.162
^2^H_3_-acetylcarnitine	1.058	432.707	689.785	0.567	0.999
^2^H_3_-propionylcarnitine	0.194	0.384	0.038	0.225	0.161
^2^H_3_-butyrylcarnitine	2.914	42.155	0.164	92.851	0.277
^2^H_9_-isovalerylcarnitine	0.217	0.519	0.103	0.215	0.173
5th percentile	0.127	0.244	0.033	0.106	0.067
Median	0.166	0.384	0.072	0.171	0.188
95th percentile	1.565	120.266	139.343	24.170	1.170

CV: coefficient of variation of all 65 samples, calculated by: standard deviation intensity/mean intensity. Median absolute variation for the most extreme storage condition was calculated by: (1) calculation of the fold change: intensity of the most extreme condition/intensity of the reference sample; (2) fold change – 1; (3) conversion to absolute numbers; (4) calculation of the median absolute variation: median of the absolute variation of the three CSF pools.

**Table 2:** 106 *m*/*z* peaks corresponding to metabolites important for neurometabolic diagnostics in the cerebrospinal fluid.

Amino Acids (24)	Neurotransmitters (21)	Purines, Pyrimidines (20)	Organic Acids (16)
Alanine Arginine	3-Methoxytyrosine/3-OMD/Methyldopa	5-Hydroxymethyluracil Adenine	n-Acetylaspartylglut. Acid 2-Methylcitric acid
Asparagine	5-Hydroxyindoleacetic acid	Adenosine/Deoxyguanosine	3-Hydroxybutyric acid
Aspartic acid	5-Hydroxytryptophan	AICAR	3-Hydroxyisovaleric acid
Cysteine	5-Methyltetrahydrofolic acid	Deoxyadenosine	4-Guanidinobutanoic acid
Cystine	Dopamine	Deoxyinosine	Acetoacetic acid
Glutamic acid	Dopamine 4-sulfate	Dihydrothymine	Cis-Aconitic acid
Glutamine	Dopamine glucuronide	Dihydrouracil	Citric acid
Glycine	Epinephrine	Guanosine	Fumaric acid
Histidine	Epinephrine glucuronide	Hypoxanthine	Lactic acid/
Homoarginine Homocarnosine	Homovanillic acid/3-OH-phenyllactic acid	Inosine Orotic acid	3-OH-propionic acid Malic acid
(Iso)leucine	Epinephrine sulfate	SAICAR	n-Acetylaspartic acid
Lysine	Gamma-aminobutyric acid	Succinyladenosine	Oxalacetic acid
Methionine	Glutamic acid	Thymidine	Propionic acid
Phenylalanine	l-Dopa/Dihydroxyphenylalan.	Thymine	Pyruvic acid
Proline	Methoxyhydroxyphenylglycol	Uracil	Succinic acid/
Serine Threonine	n-Acetylserotonin Norepinephrine	Uric acid Uridine	Methylmalonic acid
Tryptophan	Norepinephrine sulfate	Xanthine	
Tyrosine	Serotonin		
Ornithine	Vanillactic acid	**Other (1)**	**Methyldonor (2)**
Taurine	Vanillylmandelic acid	Saccharopine	s-Adenosylhomocysteine
Valine			s-Adenosylmethionine
**Creatine m. (5)**	**Vitamers (5)**	**Pterines (6)**	**Vitamin B6 vitamers (8)**
Creatine	Folic acid	7,8-Dihydroneopterin	Pipecolic acid
Creatinine	Thiamine	Dihydrobiopterin	Pyridoxal
Guanidoacetic acid Phosphocreatine	Thiamine monophosphate Thiamine pyrophosphate	Neopterin Biopterin/Sepiapterin/Primapterin	Pyridoxal 5′-phosphate Pyridoxamine
Phosphocreatinine	Thiamine triphosphate	/6-Pyruvoil-tetrahydropterin	Pyridoxamine 5′-phosph.
		Tetrahydroneopterin	Pyridoxine
		Tetrahydrobiopterin	Pyridoxine 5′-phosphate
			Alpha-aminoadipic acid delta-semialdehyde

Groups of neurometabolic metabolites are depicted in bold. Abbreviations: 3-OH: 3-hydroxy; 3-OMD: 3-O-methyldopa; AICAR: 5-aminoimidazole-4-carboxamide ribonucleotide; m: metabolism; SAICAR: succinyl-aminoimidazole-4-carboxamide ribonucleotide.

**Table 3:** Number of metabolites possibly affected by the most extreme storage condition, after correction for the analysis variability.

	Freeze/Thaw Cycle 7	−20 °C, 4 Months	5–8 °C, 168 h	18–22 °C, 168 h
Median absolute variation sILC	0.384	0.072	0.171	0.188
Range analysis variability	0.616–1.384	0.928–1.072	0.829–1.171	0.812–1.188
**1852 *m*/*z* peaks**				
*m*/*z* peaks with decreased intensities	45	142	206	67
*m*/*z* peaks with increased intensities	74	134	213	54
**106 *m*/*z* peaks corresponding to neurometabolic metabolites**				
*m*/*z* peaks with decreased intensities	3	5	8	1
*m*/*z* peaks with increased intensities	3	6	15	5

Groups of *m*/*z* peaks are depicted in bold. The median absolute variation of the sILC, as demonstrated in Table 1, reflects the variability in the sample that can be attributed to technical aspects of the analysis. The range of the analysis variability is calculated by a fold change of 1 ± median absolute variation of the sILC. *m*/*z* peaks were considered decreased when the upper limit of the 95% confidence interval of the *m*/*z* peak was below the range of the analysis variability, and increased when the lower limit of 95% confidence interval of the *m*/*z* peak was above the range of the analysis variability. Abbreviations: sILC: stable isotope-labeled compound.

## Supplementary Materials

The following are available online at https://www.mdpi.com/2218-1989/9/10/236/s1, Figure S1: Principal component analysis of the different storage conditions, Figure S2: Fold changes of *m*/*z* peaks potentially affected by multiple freeze/thaw cycles, Figure S3: Fold changes of *m*/*z* peaks potentially affected by prolonged storage at −20°C, Figure S4: Fold changes of *m*/*z* peaks potentially affected by prolonged storage at 5–8 °C, Figure S5: Fold changes of *m*/*z* peaks potentially affected by prolonged storage at 18–22 °C.
