# Supplementary material for: Assessing the Pre-Analytical Stability of Small-Molecule Metabolites in Cerebrospinal Fluid Using Direct-Infusion Metabolomics
Source: Metabolites. 2019 Oct 18;9(10):236. doi: 10.3390/metabo9100236 (PMC6835587; doi:10.3390/metabo9100236)
Supplement: Supplementary file 1 [file metabolites-09-00236-s001.pdf]

**Figure S1.** Principal component analysis of the different storage conditions

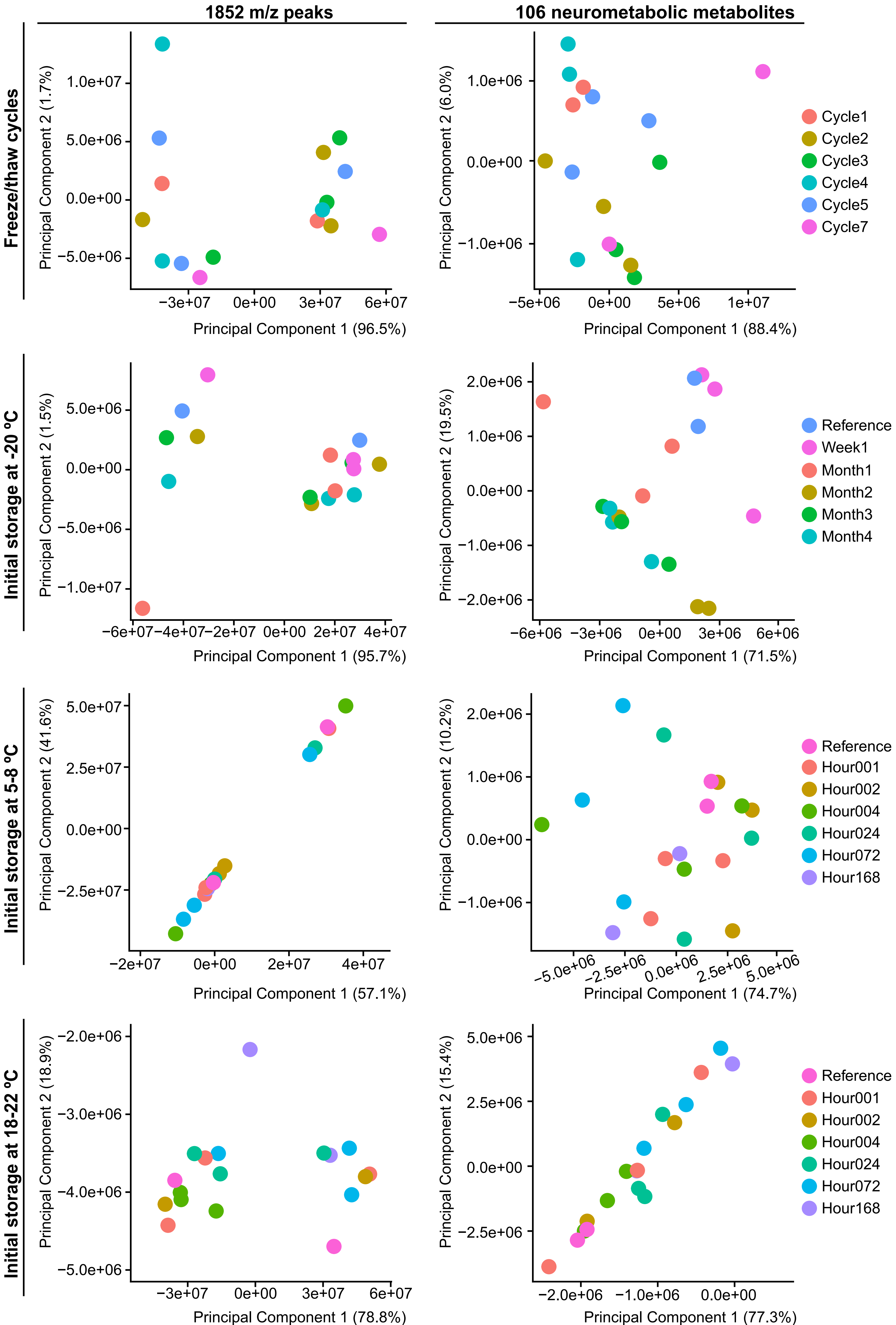

**Figure S2. Fold changes of metabolites potentially affected by multiple freeze/thaw cycles.**

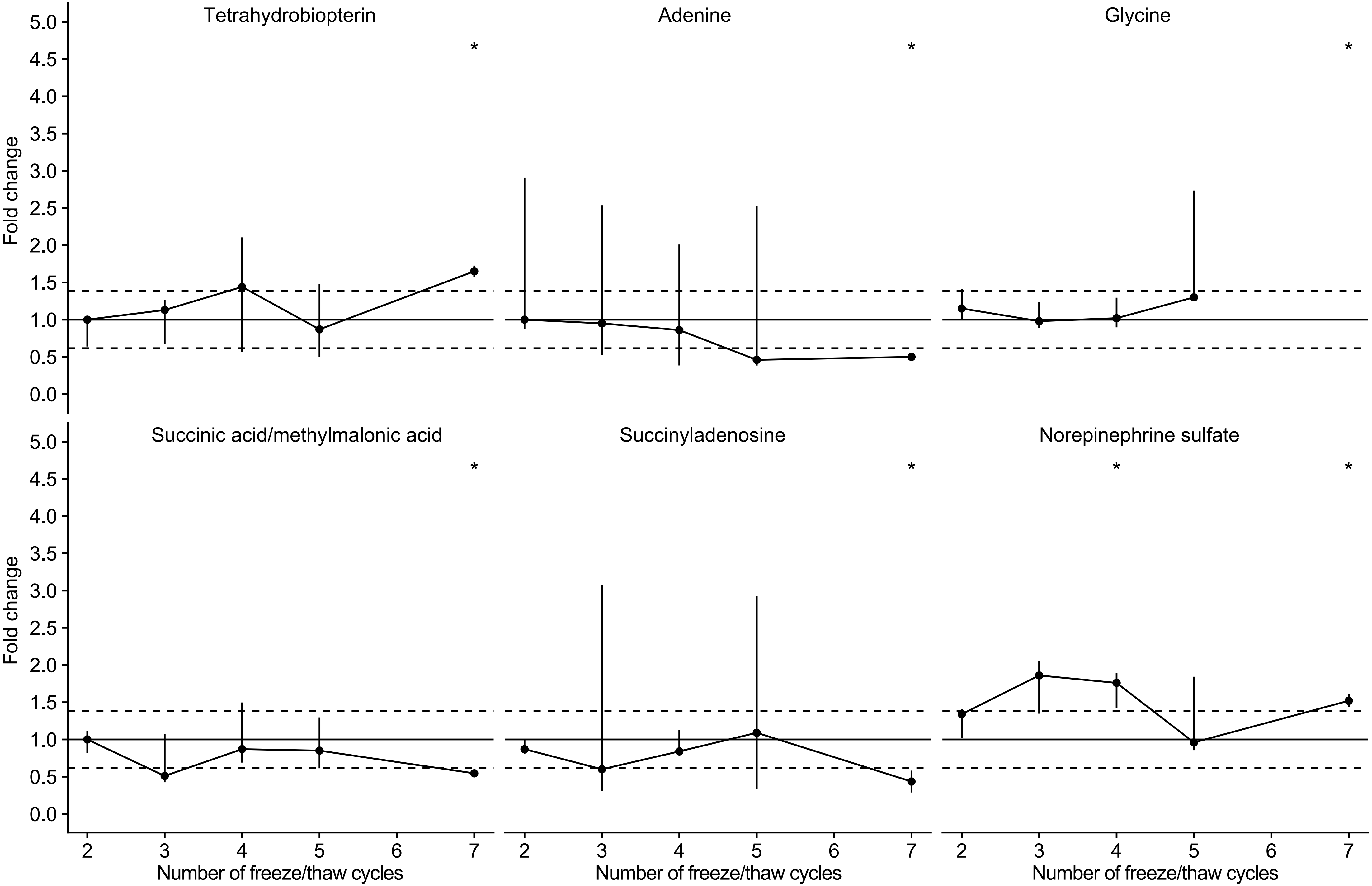


The y-axis depicts the fold change, the x-axis depicts the number of freeze/thaw cycles. The filled black circle depicts the mean fold change, the line depicts the range of the confidence interval. The horizontal black line depicts fold change =1, horizontal dashed lines indicate the variability that is solely attributed to the analysis, as in Figure 2. Asterisks depict the storage condition for which the lower limit of the confidence interval was above the upper dashed line, and for which the upper limit of the confidence interval was below the lower dashed line.

**Figure S3. Fold changes of metabolites potentially affected by prolonged storage at −20 ºC .**

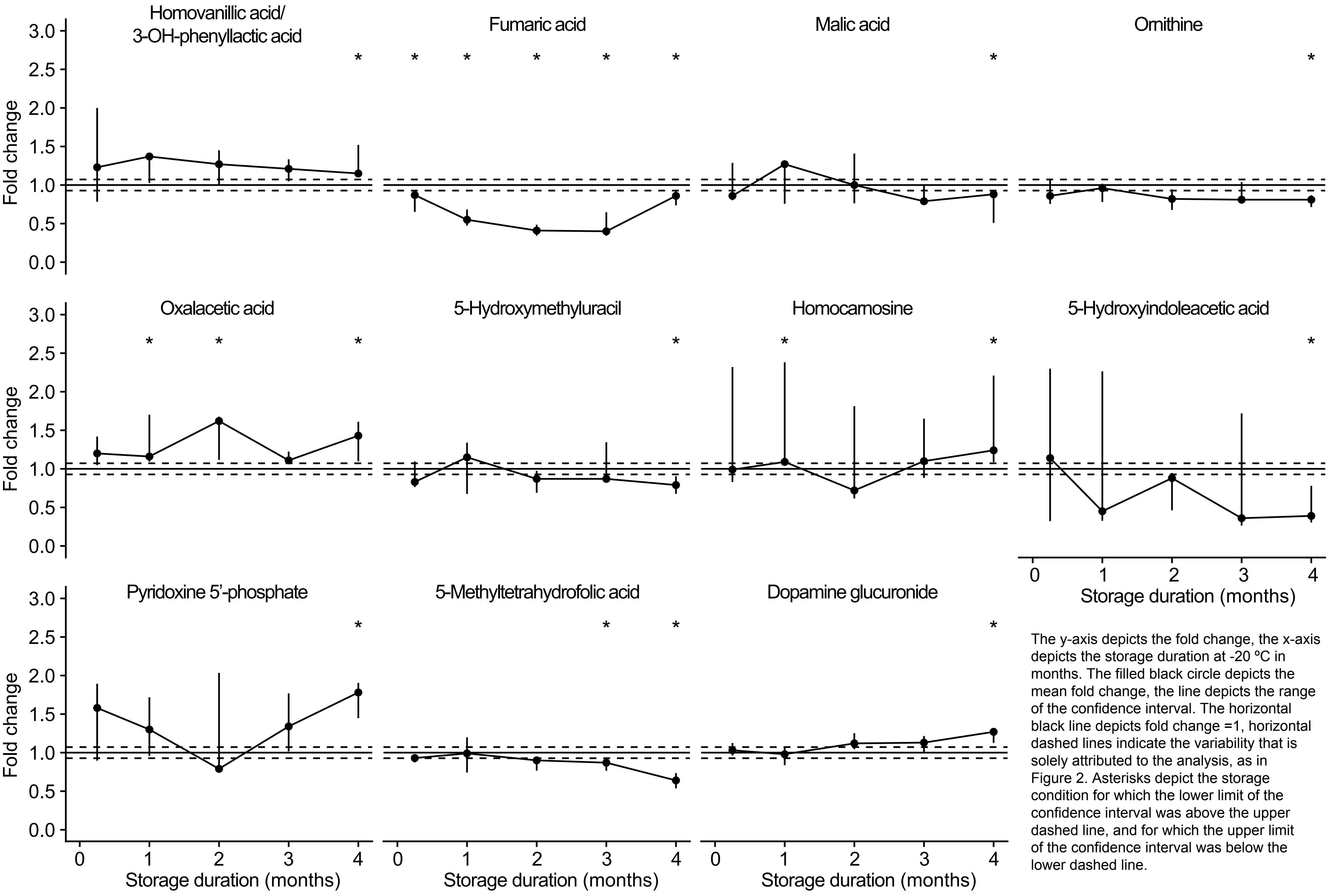


The y-axis depicts the fold change, the x-axis depicts the storage duration at -20 ºC in months. The filled black circle depicts the mean fold change, the line depicts the range of the confidence interval. The horizontal black line depicts fold change =1, horizontal dashed lines indicate the variability that is solely attributed to the analysis, as in Figure 2. Asterisks depict the storage condition for which the lower limit of the confidence interval was above the upper dashed line, and for which the upper limit of the confidence interval was below the lower dashed line.

**Figure S4. Fold changes of metabolites potentially affected by prolonged storage at 5-8 ºC .**

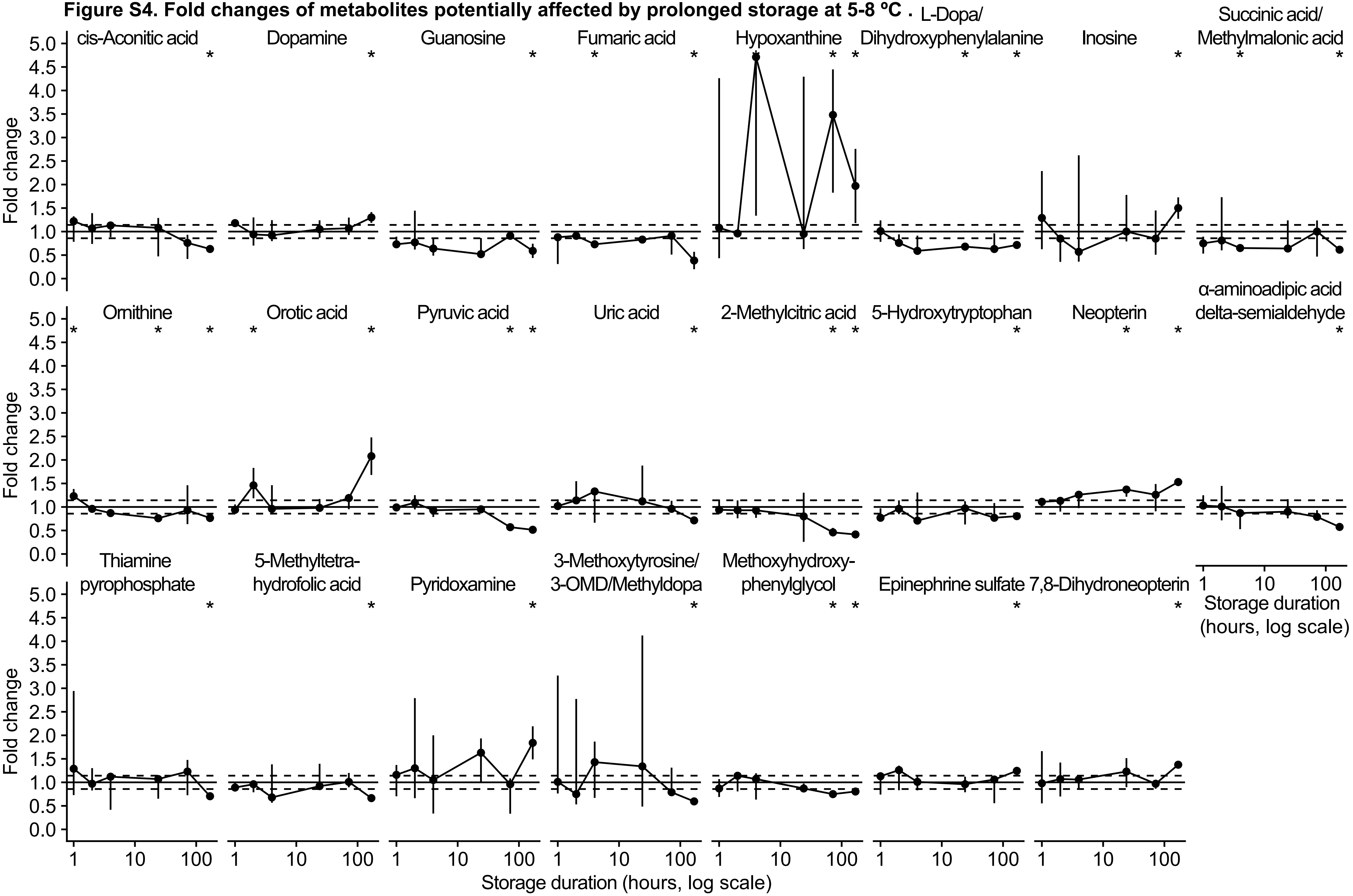


The y-axis depicts the fold change, the x-axis depicts the storage duration at 5-8 ºC in hours. The filled black circle depicts the mean fold change, the line depicts the range of the confidence interval. The horizontal black line depicts fold change =1, horizontal dashed lines indicate the variability that is solely attributed to the analysis, as in Figure 2. Asterisks depict the storage condition for which the lower limit of the confidence interval was above the upper dashed line, and for which the upper limit of the confidence interval was below the lower dashed line.

**Figure S5. Fold changes of metabolites potentially affected by prolonged storage at 18-22 ºC .**

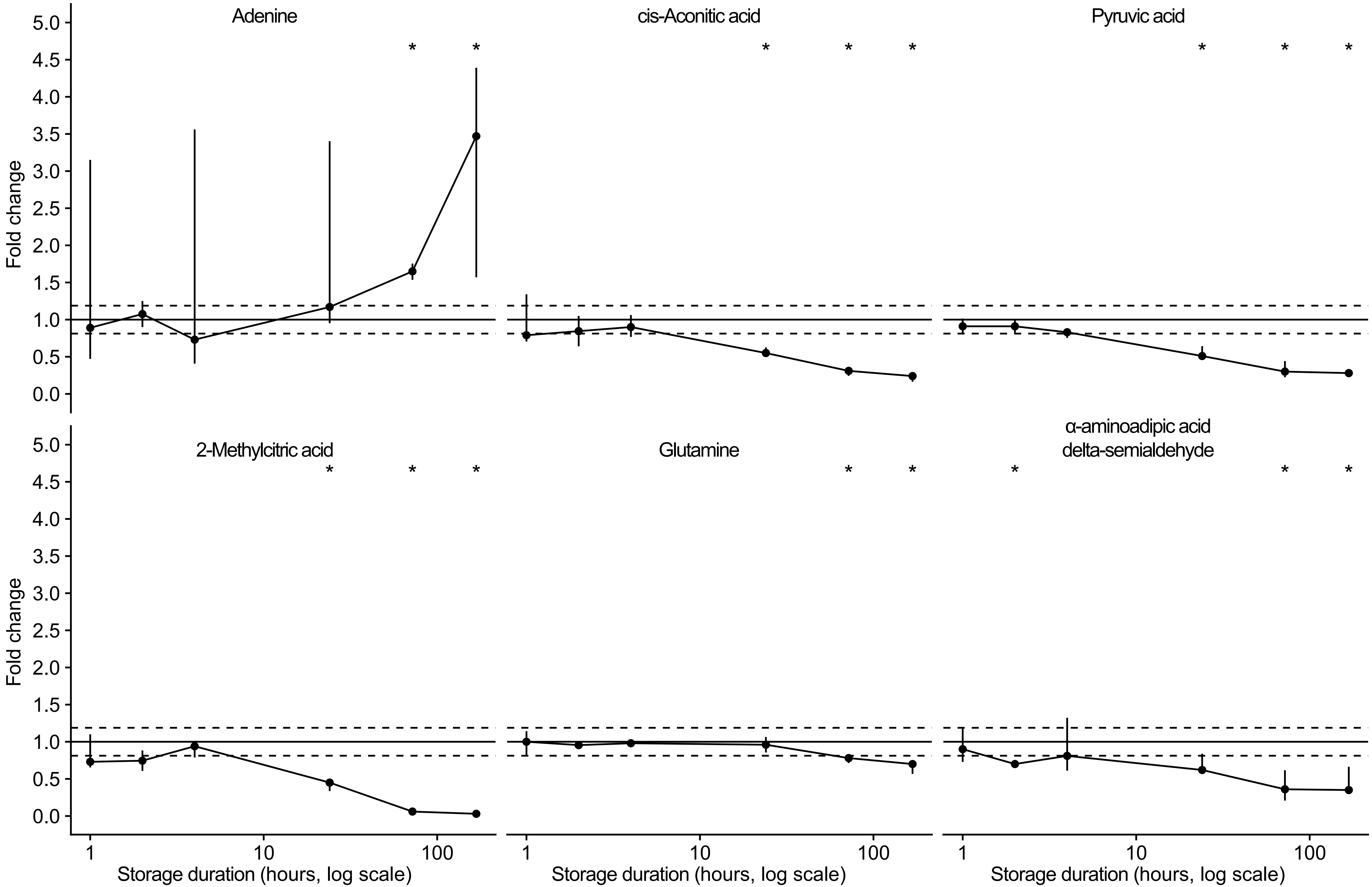


The y-axis depicts the fold change, the x-axis depicts the storage duration at 18-22 ºC in hours. The filled black circle depicts the mean fold change, the line depicts the range of the confidence interval. The horizontal black line depicts fold change =1, horizontal dashed lines indicate the variability that is solely attributed to the analysis, as in Figure 2. Asterisks depict the storage condition for which the lower limit of the confidence interval was above the upper dashed line, and for which the upper limit of the confidence interval was below the lower dashed line.
